# Protein binding of vancomycin in a large mixed patient population at a university hospital

**DOI:** 10.1128/aac.01593-25

**Published:** 2026-01-26

**Authors:** Alexander Dejaco, Constantin Lier, Sabrina Krautbauer, Frieder Kees, Christoph Dorn, Martin G. Kees

**Affiliations:** 1 Department of Anaesthesiology, University Hospital Regensburg 39070, Regensburg, Germany; 2 Department of Pharmaceutical and Medicinal Chemistry I, University of Regensburg 199897, Regensburg, Germany; 3 Institute of Clinical Chemistry and Laboratory Medicine, University Hospital Regensburg 39070, Regensburg, Germany; 4 Department of Pharmacology, University of Regensburg 9147, Regensburg, Germany; Providence Portland Medical Center, Portland, Oregon, USA

**Keywords:** protein binding, immunoassay, antibiotic, HPLC, ultrafiltration, unbound fraction, therapeutic drug monitoring

## Abstract

Vancomycin remains a key treatment for infections caused by β-lactam-resistant gram-positive cocci. While there is unanimity that only drug not bound to plasma proteins is pharmacologically active, a wide range of values and interdependencies for the unbound fraction (*f*_u_) of vancomycin have been reported in the past. In the present study, we evaluated 706 plasma samples from 228 adult in-patients who were sent for therapeutic drug monitoring. Total and free concentrations of vancomycin were analyzed by a validated method using ultrafiltration and HPLC-UV. Covariate effects on *f*_u_ were assessed by a linear mixed-effects model. The mean unbound fraction was 72.2 ± 5.5% (coefficient of variation 7.7%, range 53–93%), the intra-individual and inter-individual variability were low (median coefficient of variation 5.7% and 6.4%, respectively). The unbound fraction was independent of total vancomycin, plasma albumin or total protein concentrations, or other biochemical or demographic variables, and did not differ between patients treated inside or outside of intensive care (*P*=0.465). Linear mixed-effects modeling confirmed low overall variability (coefficient of variation 7.0%), decomposed into 2.2% inter-individual, 3.8% inter-occasion, and 5.5% residual variability. Method comparison showed an excellent agreement between high-performance liquid chromatography with ultraviolet detection (HPLC-UV) and the immunoassay used for routine drug monitoring (bias +0.2%). Free concentrations of vancomycin can be reliably predicted from total concentrations. An unbound fraction of approximately 70% provides a robust and clinically useful estimate. Remaining variability appears to be primarily methodological, and not of clinical relevance.

## INTRODUCTION

Despite the development of novel anti-staphylococcal agents, vancomycin (VAN) remains widely used to treat infections caused by methicillin-resistant *Staphylococcus aureus* (MRSA) and other β-lactam-resistant gram-positive cocci. Its use has increased since the epidemic of MRSA began in the 1990s (1, 2). However, its safe and effective use requires therapeutic drug monitoring (TDM) (3). Routine TDM of vancomycin involves measuring total drug concentrations by immunological assays using trough concentrations or, preferably, the area under the curve (AUC)/MIC ratio as pharmacokinetic/pharmacodynamic (PK/PD) target (4–6). Regardless of the chosen target, it is important to recognize that all PK/PD indices should be referenced to the unbound (non-protein bound) fraction of the drug (7). A protein binding of 50% is often used to calculate free vancomycin concentrations, albeit the protein binding of vancomycin is showing considerable inter-patient variability across studies (ranging from almost 0 to 90%) (8). As high variability of protein binding can distort the prediction of the free concentration, the direct measurement of free concentrations has been recommended (9–11).

Equilibrium dialysis is generally accepted as the ‘gold standard’ for plasma protein binding studies in drug discovery and drug development (12, 13). But ultrafiltration is by far the most popular approach in the clinical setting, where the free concentrations are to be addressed, and the degree of protein binding or *f*_u_ is only of academic interest (14). While with ultrafiltration, the free concentration is directly measured, with equilibrium dialysis, the free concentration is calculated from three separate measurements: the concentration in the buffer chamber and in the plasma chamber at equilibrium, which gives *f*_u_, and the measured total concentration in the *ex vivo* sample, which is multiplied by *f*_u_ to obtain the free concentration. This complex procedure makes equilibrium dialysis practically unsuitable for routine clinical use (15).

Although direct measurement of the free concentrations would be preferable, this is not widely used in clinical practice for several reasons, including analytical pitfalls (14).

We have developed an ultrafiltration method which, like equilibrium dialysis, mimics physiological conditions, including control of pH, and which was successfully applied to the determination of free concentrations of vancomycin in patients treated in the intensive care unit (ICU) (16). This method was further refined and validated in as much as 30 drugs, mostly antimicrobials (15).

The aim of the present study was to apply the method to a large number of samples from clinical routine TDM for vancomycin in order to (i) evaluate the suitability of the ultrafiltration method for routine use and (ii) to further gain insight into the variability of the protein binding and the unbound fraction of vancomycin. A secondary aim was to compare the total concentrations of vancomycin as measured by high-performance liquid chromatography with ultraviolet detection (HPLC-UV) with the routine clinical immunological assay.

## RESULTS

### Patients and data basis

A total of 768 plasma samples from 231 adult patients were collected between April and August 2023. Thirty-one samples were excluded due to insufficient plasma volume. Three samples were excluded, as the HPLC determination of free vancomycin was interfered with (UV spectrum did not match with vancomycin). Fifteen patients (comprising 96 samples) were transferred between two wards during the vancomycin therapy. These cases were assigned to the ward providing the majority of samples (in sum *n* = 68; the remaining 28 samples from the secondary wards were excluded). In sum, 62 plasma samples were excluded, and 706 plasma samples from 228 patients were evaluated (see Table 1 for descriptive statistics; an overview of all missing data can be found in Fig. S1). Information regarding the clinical specialty was available for all subjects. The list of ward specialties and their respective observation counts is presented in Table S1. For 91 patients only one sample was available; for the remaining 137 patients, 2–17 (median 4) samples.

**TABLE 1:** Demographic and laboratory data (median, range, or percentage for sex) and corresponding number of samples [patients] of in-patients for whom vancomycin TDM was available^*a*^

	Value	No. of samples [from no. of patients]
Age (years)	63 (18–86)	706 [228]
Sex (m/f)	68/32%	480 [154]/226 [74]
Creatinine (mg/dL)	1.08 (0.23–7.9)	670 [220]
eGFR (mL/min)	66 (4–194)	643 [218]
Total bilirubin (mg/dL)	0.80 (0.20–31.1)	540 [209]
Total protein (g/L)	56.4 (29.2–92.3)	92 [70]
Albumin (g/L)	30.2 (15.3–47.5)	387 [156]
‍IgA (g/L)	2.03 (1.51–4.01)	12 [9]
CRP (mg/L)	64.4 (0.6–438)	678 [225]
Leukocytes (1/µL)	8,355 (0–93,910)	676 [225]
‍Hemoglobin (g/dL)	8.8 (4.0–16.5)	675 [73]
VAN_IA_ (mg/L)	14.9 (4.17-65.1)	706 [228]

^*a*^ m: male, f: female, eGFR: estimated glomerular filtration rate using the CDK-EPI formula, CRP: c-reactive protein, and VAN_IA_: total vancomycin plasma concentrations determined by routine immunoassay (TDM).

### Concentrations and unbound fraction of vancomycin

**Fig 1:**
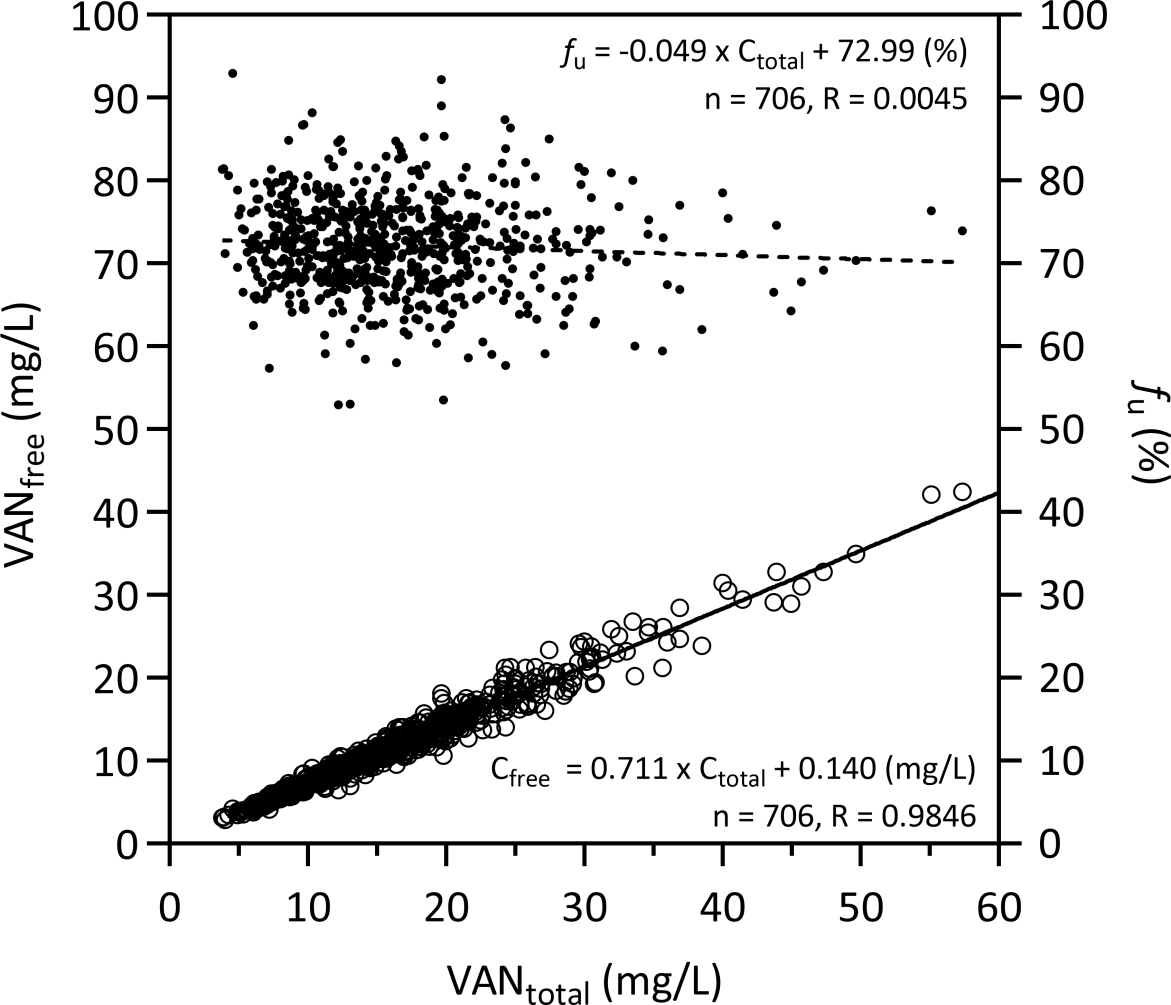
Correlation between free and total vancomycin plasma concentrations (circles, left *y*-axis) or unbound fraction and total vancomycin plasma concentrations (dots, right *y*-axis) in 706 plasma samples from 228 patients.

**Fig 2:**
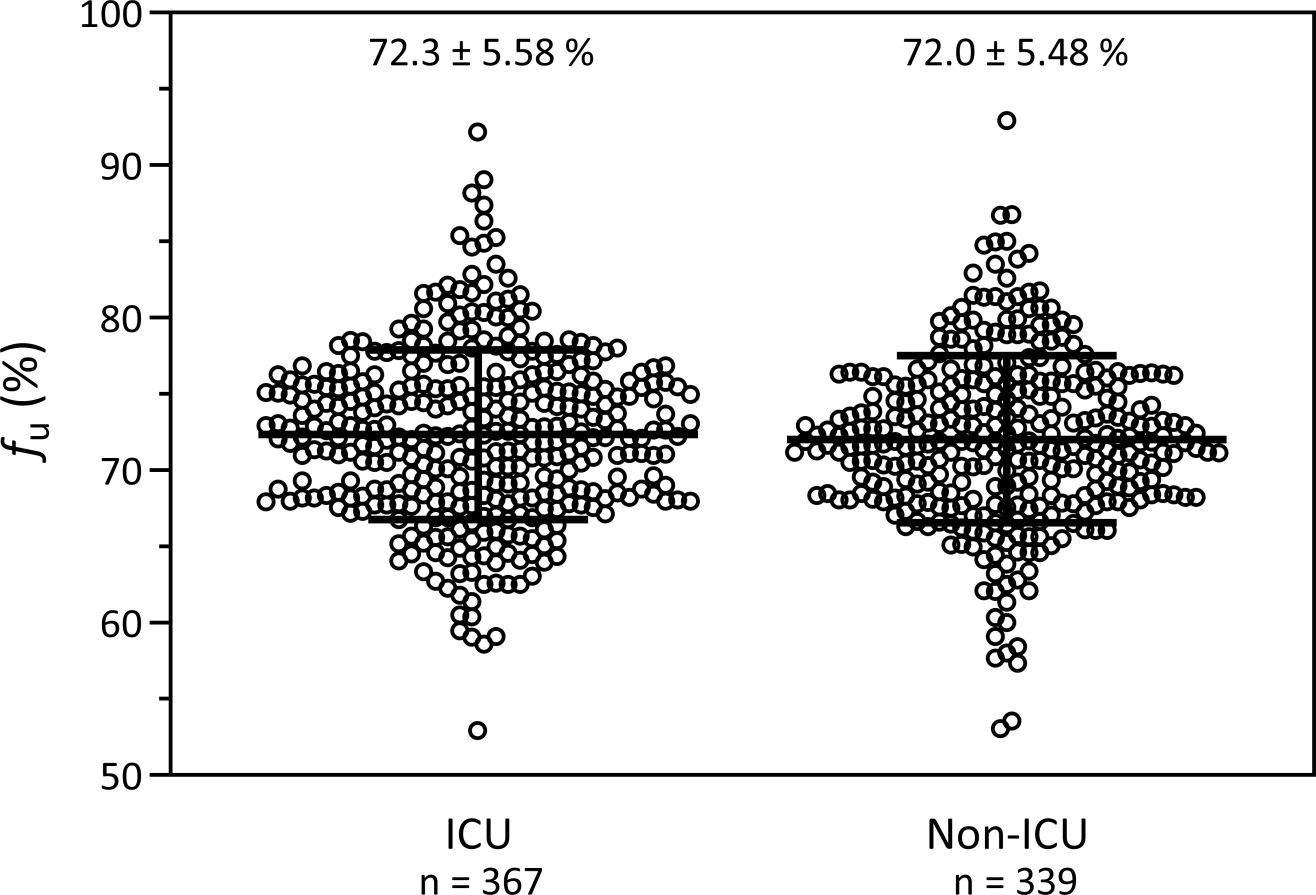
Distribution of unbound vancomycin fractions (*f*_u_, mean ± standard deviation) in patients treated in an intensive care unit (ICU; 367 samples from 96 patients) or outside of an ICU (non-ICU; 399 samples from 132 patients).

In a pooled analysis of all measurements, there was a strong linear correlation between the measured free concentrations and the total concentrations of vancomycin (*R* = 0.9846, Fig. 1 left *y*-axis). *f*_u_ of vancomycin was independent of the concentration (Fig. 1, right *y*-axis); the slope of the linear regression of *f*_u_ versus the concentration (*R* = 0.0045) was not different from zero (95% confidence interval [CI] −0.1033 to 0.0048). The mean *f*_u_ of all samples (*n* = 706) was 72.2 ± 5.5% (coefficient of variation [CV] 7.7%, range 53 to 93%; Table 2). When the mean values of the individual patients were used to ensure equal weighting of each patient (*n* = 228), the mean *f*_u_ was 72.2 ± 4.6% (CV 6.4%, range 53 to 84%). The intra-individual variability of *f*_u_ among the 73 patients with at least four plasma samples available (to allow for a reasonable calculation of CV) was 5.3% (median CV, range 1.1 to 14.5%) and was also not significantly different (*P* = 0.301) between patients with a short sampling period (first quartile, 4–7 days, median 6 days, *n* = 19, median CV 4.0%) and patients with a long sampling period (fourth quartile, 22–68 days, median 33 days, *n* = 17, median CV 5.3%). The distribution and the time courses of the *f*_u_ in these 17 patients are depicted in Fig. S2 and S3. *f*_u_ was not different between patients treated in the ICU and patients treated outside of the ICU (*P* = 0.465; Table 2; Fig. 2). *f*_u_ of patients stratified by primary ward specialty is depicted in Fig. S4.

**TABLE 2:** Total and free concentrations of vancomycin in plasma samples of ICU and non-ICU patient populations determined by HPLC and calculated unbound fraction (median, range)^*a*^

	All	ICU	Non-ICU‍
No. of samples [patients]	706 [228]	367 [96]	339 [132]‍
VAN_total_ (mg/L)	14.9 (3.85–57.4)	16.6 (4.94–45.7)	‍13.9 (3.85–57.4)
VAN_free_ (mg/L)	10.8 (2.86–42.4)	11.8 (3.44–32.8)	9.98 (2.86–42.4)‍
*f*_u_ (%)	72.1 (52.9–92.9)	72.3 (52.9–92.2)	‍71.8 (53.1–92.9)

^*a*^ VAN_total_: total vancomycin plasma concentrations, VAN_free_: free vancomycin plasma concentrations, and ICU: intensive care unit.

### Correlation of unbound fraction with plasma proteins

**Fig 3:**
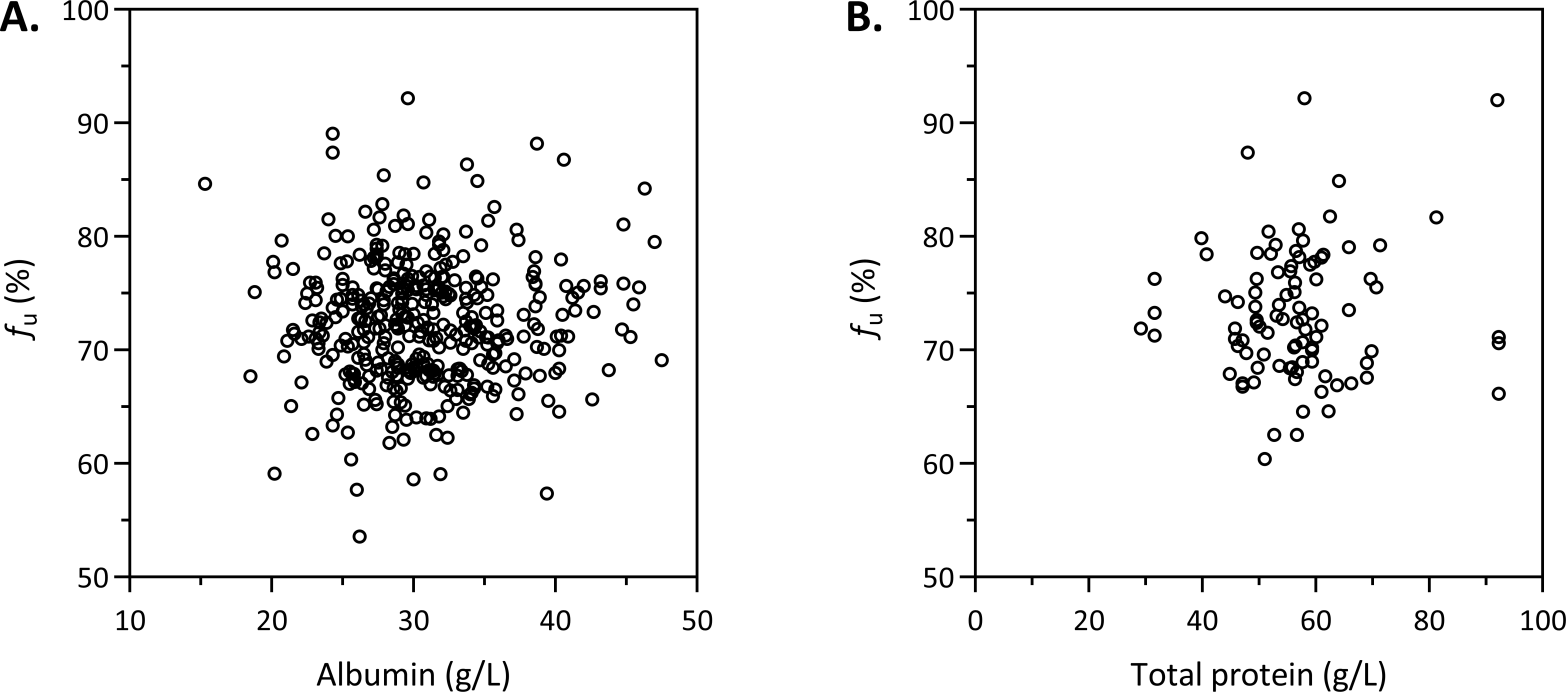
Scatter plot of unbound fraction (*f*_u_) against (**A**) albumin (387 plasma samples from 156 patients) or (**B**) total protein concentration (92 plasma samples from 70 patients).

The concentrations of albumin were available for 156 patients (387 samples), while the total protein concentrations were available for 70 patients (92 samples, Table 1). As shown in Fig. 3, no correlation was found between the unbound fraction and the albumin or total protein concentration in the pooled analysis. The IgA plasma concentration was available for eight patients; in seven patients, the concentrations were 1.65–2.29 g/L; and, in one patient (4.0 g/L), it was at the upper limit of the reference range (0.70–4.0 g/L). *f*_u_ in these patients was 72.4 ± 4.0% and 68.7% in the patient with the highest IgA concentration.

### Linear mixed-effects modeling

The initial complete-case covariate model was limited to 49 observations from 40 subjects due to missing data across several covariates, with the overlap of albumin and total protein measurements being particularly small (Fig. S1). This overlap was insufficient to support a meaningful model. After excluding total protein, the model could be fitted using 284 observations from 138 subjects across 189 occasions. Following removal of non-significant fixed effects, the final model included all 706 observations from 228 subjects and 330 occasions. Buffer type was strongly associated with *f*_u_ (*P* < 0.001): phosphate buffer increased *f*_u_ by an estimated 5.8% compared with HEPES buffer (95% CI 4.9 to 6.7). Ward specialty also contributed significantly overall (*P* = 0.013). Post hoc pairwise comparisons of estimated marginal means indicated that *f*_u_ in vascular surgery (66.9%, 95% CI +63.8 to +69.9) was significantly lower than in neurosurgery (73.5%, 95% CI 71.4 to 75.6, adjusted *P* = 0.030), while all other contrasts lost significance after multiple testing correction. Model predictions across ward specialty are shown in Fig. S5. The model revealed an overall CV for *f*_u_ of 7.0% composed of a CV of 2.2% for inter-individual, 3.8% for inter-occasion, and 5.5% for residual “unknown” variability (i.e., an intra-class correlation coefficient of 39.6%). Model specifications and full model results are provided in Table S2.

### Method comparison

**Fig 4:**
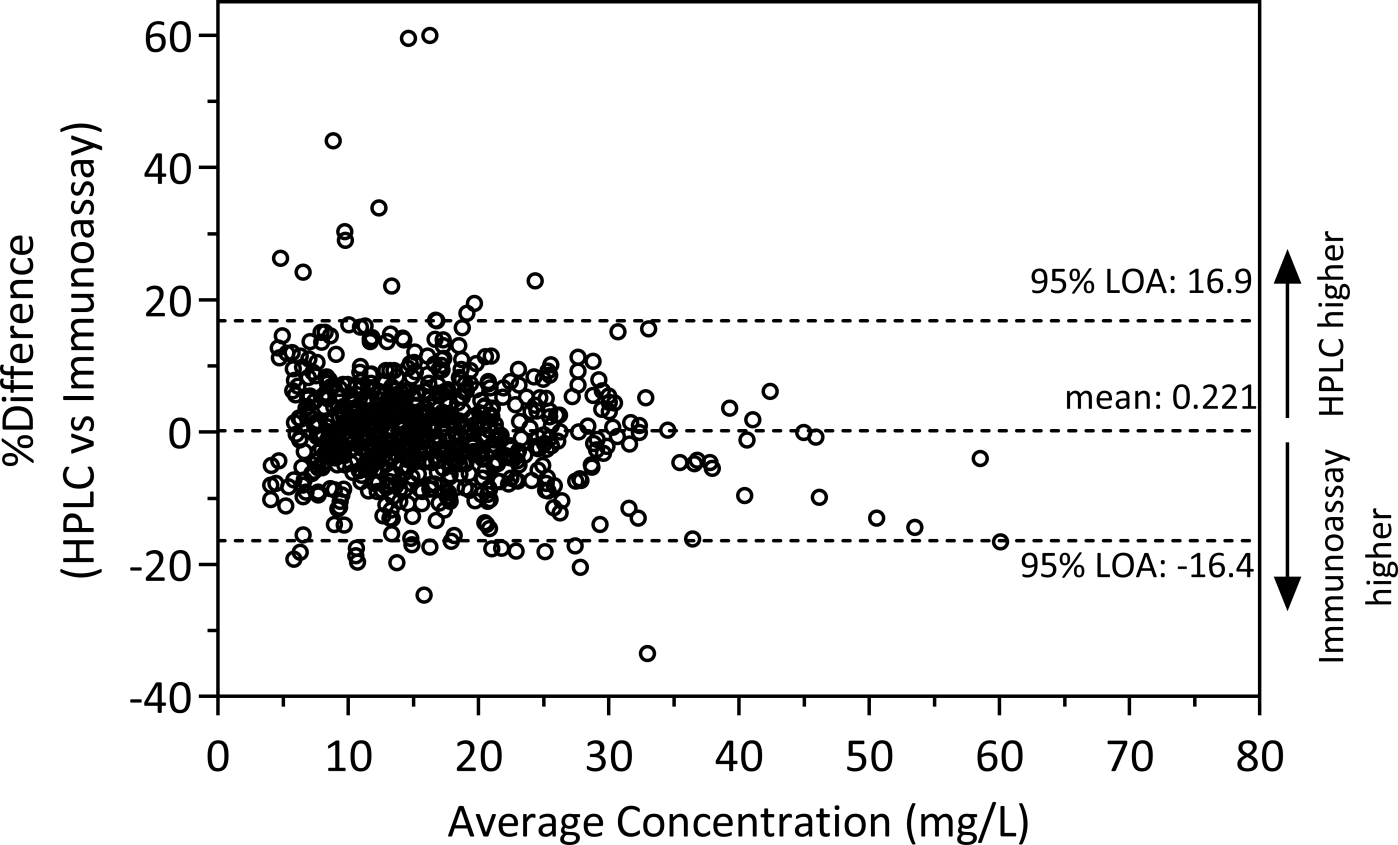
Bland-Altman plot showing the percentage difference between HPLC-measured and immunoassay-measured total vancomycin concentrations against their average concentration. Horizontal dashed lines indicate the mean difference and the 95% limits of agreement (LOA).

Total vancomycin concentrations as measured by HPLC or immunoassay showed virtually no bias (+0.221%) and good agreement (limits of agreement −16.4 to 16.9%) (Fig. 4). The concentrations as measured by HPLC tended to be lower at immunoassay concentrations > 40 mg/L and were in few cases markedly higher at concentrations <20 mg/L (Fig. S6).

## DISCUSSION

Numerous studies have investigated the extent and variability of vancomycin protein binding in humans since the first report in 1966, which indicated a protein binding of less than 10% determined by equilibrium dialysis. In the 1980s, protein binding between 42 and 55% was reported using ultrafiltration or equilibrium dialysis (17–19). Between 1988 and 1993, a number of studies found about 30% protein binding (20–26). This value of 30% was included in the pharmacological reference textbook *Goodman & Gilman’s The Pharmacological Basis of Therapeutics* (27). In the current SPC, a range of 30–55% is specified (28).

Possibly triggered by a case report of extensive vancomycin binding in a patient with IgA myeloma (97–98% at IgA of 46 g/L and 92–93% at IgA of 8-10 g/L), higher mean protein binding (36–55%) and high intra- and inter-individual variabilities have been reported in several studies comprising 15–84 patients (9, 10, 29–32). Studies investigating the possible influence of various plasma proteins, which may explain the high variability of vancomycin protein binding, gave inconsistent and contradictory results (8, 10, 24, 26, 33–35).

The higher binding rates and the high variability are in contrast to a valuable study in 22 patients, which not only determined a lower protein binding rate and lower variability (25.6 ± 6.6%, corresponding to *f*_u_ of 74.4 ± 6.6%, CV 8.9%) as assessed by ultrafiltration, but also demonstrated equivalence of ultrafiltration with modern fast micro-equilibrium dialysis (28.0% ± 5.7%, corresponding to *f*_u_ of 72.0 ± 5.7%, CV 7.9%) as reference method (36). No significant influence of plasma proteins, such as albumin, total protein, or alpha1-acid glycoprotein, on the protein binding of vancomycin was found, and an only moderately increased protein binding (37%) was measured in a patient plasma sample with high IgA concentrations (33.8 g/L).

The present study confirms these results in a large cohort of 228 patients and provides clarity on the extent and the variability of the unbound fraction of vancomycin in adult patients. We found (i) a good linear correlation between free and total concentrations, (ii) a mean *f*_u_ of 72% (i.e., a protein binding of 28%), with a (iii) low intra-individual variability (CV < 15%) as well as a low inter-individual variability (CV 6.4%), and (iv) no influence of several clinical covariates, such as demographic or biochemical parameters on *f*_u_. Furthermore, *f*_u_ did not differ between ICU- and non-ICU patients. Linear mixed-effects modeling found a statistically significant difference in *f*_u_ between patients in vascular surgery (66.9%) and those in neurosurgery (73.5%, adjusted *P* = 0.030) in post hoc comparisons; however, this finding is not clinically relevant and should not be overinterpreted. It should be noted that incomplete covariate data reduced the number of observations available for modeling, limiting the statistical power to detect weak associations (Fig. S1).

Against this background, concerns arise regarding the reliability of some previous studies, particularly when considering the influence of experimental conditions during ultrafiltration (9, 10, 30, 32, 37). Reported protocols either involved centrifugation at low temperature or yielded inconsistent results depending on the molecular weight cut-off of the membrane and the specific centrifugation conditions, or a hollow fiber membrane (polysulfone) prone to adsorption was used (9, 30, 32, 37). Each of these experimental conditions will result in falsely low and presumably more variable free concentrations. Analytical imprecision is also a plausible explanation for the poor correlation between free and total vancomycin concentrations reported in some studies, which is in contrast to other studies including the present one (8–10, 30, 31, 36).

Moreover, the substantial intra-individual variability of *f*_u_, as found in a previous study comprising seven patients (estimated CV ranging from 10 to 70%), which was mainly attributed to the long treatment duration (up to 41 days) (9), could not be observed in our extensive study, where intra-individual variability was low (CV ranging from 1 to 15% in *n* = 73 patients with at least four plasma samples available) and independent of the length of the sampling period (up to 68 days). The linear mixed-effects model decomposed the estimated overall CV of 7.0% into an inter-individual CV of only 2.2%, an inter-occasion CV of 3.8%, and a residual 'unknown' variability of 5.5%, which may reflect (low) analytical imprecision.

As outlined in the Introduction, ultrafiltration is the most widely used method for measuring the free concentrations and determining *f*_u_, yet it is also a major source of error and variability due to its dependence on experimental conditions (15, 36, 38). Moreover, since *f*_u_ is derived from both total and free concentration measurements, its analytical variability reflects the propagated imprecision of the two independent assays.

With respect to the measurement of total concentrations, our study showed good agreement between the HPLC-PDA-based method and the VANC3 immunoassay used in clinical routine, which is based on the kinetic interaction of microparticles in solution (KIMS). Regarding the measurement of free concentrations, it should be noted that immunoassays are generally not validated for ultrafiltrate. Matrix effects arising from the use of different sample matrices (protein-containing plasma versus protein-free ultrafiltrate) may contribute to analytical variability (9, 32, 39).

### Limitations

The ultrafiltration method as used in the present study was originally developed for the determination of free vancomycin and contained phosphate buffer to maintain a physiological pH during ultrafiltration (16). Based on a concurrently conducted methodological study with 30 drugs using spiked serum of healthy volunteers, the standard protocol was modified during the course of the present study. Phosphate was replaced by HEPES, which is apparently more widely applicable and does not interfere with polyvalent metal ions, such as calcium (15). For the same reason, the International Federation of Clinical Chemistry International Conventional Reference Procedure for the measurement of free thyroxine in serum recommends HEPES instead of phosphate for the determination of free thyroxine by equilibrium dialysis (40, 41). In the abovementioned methodological study, *f*_u_ of vancomycin was 78.0 (unbuffered), 77.4 (phosphate), or 74.0% (HEPES) (15). In the study of Stove et al. comprising 39 samples from 22 patients, *f*_u_ was 74.4 ± 6.6% as determined by ultrafiltration of unbuffered plasma and 72.0 ± 5.7% as determined by equilibrium dialysis using HEPES (38). In the present study, *f*_u_ in patients was 73.6 ± 7.8% using phosphate or 68.1 ± 4.1% using HEPES and 69.6 ± 1.7% or 66.7 ± 2.9% in spiked control samples (cf. Materials and Methods). Although *f*_u_ tended to be lower with HEPES, the differences were small and within the range of imprecision in clinical routine. Apparently, buffering or the type of buffer appears to be of minor importance in the case of vancomycin (36). However, as *f*_u_ of vancomycin increases with high pH, buffering can become relevant when using stored serum samples, during which the pH may rise to pH >8.5 (16). From a modeling perspective, incomplete covariate information for a subset of samples led to a reduction in the effective sample size for the mixed-effects analysis, thereby potentially reducing the statistical power to detect weak predictors. However, given the overall still substantial cohort size and the consistently low variability of the unbound fraction across repeated analyses, this limitation is unlikely to significantly affect the validity of the results.

### Conclusion

The present study confirms in a large set of 228 adult patients the results of a smaller study in 22 patients (38). The free concentrations of vancomycin can be reliably predicted from the total concentrations. A value of 70% is a good estimator for the unbound fraction of vancomycin in clinical practice. The high variability of the unbound fraction or the weak correlation between total and free concentrations as reported in the literature is mainly attributable to methodological shortcomings and analytical imprecision. There is now little unexplained variability left, which we feel is not worthy of further clinical research.

## MATERIALS AND METHODS

### Patient population and samples

Samples were analyzed by routine immunoassay, then kept up to 6 days at 4–8°C and subsequently stored at −70°C until HPLC analysis. Non-identifying demographic (age, sex) and biochemical parameters as well as the submitting ward were extracted from the laboratory information system. Hospital wards were categorized as either ICU or non-ICU, with the latter further sub-classified according to their primary specialty. Human serum from healthy volunteers was used for preparing calibrator and quality control samples for HPLC analysis.

### Drugs and chemicals

Vancomycin-HCl (secondary analytical standard) was obtained from Sigma-Aldrich (Taufkirchen, Germany); acetonitrile, methanol (both HPLC gradient-grade), and the other chemicals were obtained from VWR (Darmstadt, Germany). HPLC-grade water was produced using an Arium basic water purification system (Sartorius, Göttingen, Germany). Vivafree 500 30 kD Hydrosart centrifugal ultrafiltration devices were obtained from Vivaproducts, Inc. (Littleton, MA, USA).

### Clinical chemistry

Biochemical analyses (creatinine, total bilirubin, albumin, total protein, and C-reactive protein [CRP]) in human serum and plasma were performed on a Roche Cobas C 503 System (Roche Diagnostics GmbH, Mannheim, Germany) following the manufacturer’s instructions. Creatinine was quantified using the Roche enzymatic assay based on sequential enzymatic conversion of creatinine and subsequent photometric detection of the quinone-imine chromogen. Total bilirubin was measured by a colorimetric diazo method using 3,5-dichlorophenyl diazonium to form a red azo dye. Albumin concentrations were determined by bromcresol green (BCG) dye-binding with photometric readout. Total protein was measured using the biuret reaction, in which proteins form a purple copper complex under alkaline conditions. CRP was quantified by a particle-enhanced immunoturbidimetric assay using latex-bound monoclonal anti-CRP antibodies. Hemoglobin was measured using the sodium lauryl sulfate (SLS) method, and leukocyte counts were obtained by impedance/flow cytometry using a Sysmex analyzer (Sysmex Deutschland GmbH, Norderstedt, Germany).

### Drug analysis by immunoassay

Total vancomycin was measured at the Department of Clinical Chemistry using the Vancomycin Gen.3 (VANC3) assay, a particle-enhanced turbidimetric immunoassay based on the Kinetic Interaction of Microparticles in a Solution (KIMS) performed with a Cobas C 503 analyzer (both Roche Diagnostics GmbH, Mannheim, Germany). According to the manufacturer, the assay is linear in plasma between 4.0 and 80 mg/L, with 4 mg/L being the lower limit of quantitation (i.e., the lowest analyte concentration that can be reproducibly measured with a total error of 20%).

### Drug analysis by HPLC

Vancomycin 5 g/L stock solution was prepared in water and stored in aliquots at −70°C. Standard samples at 20 mg/L and quality control (QC) samples at 25 (high) or 5.0 mg/L (low) were prepared by diluting the stock solution with water and finally 1:20 with blank serum for the determination of vancomycin in serum or with saline for the analysis in ultrafiltrate. Sample treatment for the determination of total vancomycin was performed as described previously with minor modifications (40). Serum/plasma (100 µL) was mixed with o-phosphoric acid (0.1 mol/L, 200 µL) and acetonitrile (500 µL). After separation of the precipitated proteins and extraction of acetonitrile into dichloromethane (1.3 mL), an aliquot (1 µL) of the upper, aqueous layer was injected into the column. The free concentrations of vancomycin in plasma were determined after ultrafiltration as recently described, including prior validation of membrane integrity and assessment of nonspecific binding (15). In brief, 10 μL 3 M potassium phosphate (pH 7.4) or 10 µL 2 M HEPES (pH 7.5 at room temperature) was pipetted into the ultrafiltration device (Vivafree 500 30 kD Hydrosart, Vivaproducts, Inc., Littleton, MA, USA) and mixed with 300 μL serum/plasma. The sample was incubated in the ultrafiltration device for 10 min at 100 × *g*/37°C for temperature adjustment (centrifuge 5417R with fixed-angle rotor 45-30-11, Eppendorf, Hamburg, Germany), and then centrifuged for 20 min at 1,000 × *g*/37°C. An aliquot (1 µL) of the ultrafiltrate (70–90 µL) was injected into the column. After ultrafiltration, the pH (at room temperature) in the remaining plasma in the upper container of the ultrafiltration devices was 7.54 ± 0.12 (*n* = 492) using phosphate and 7.64 ± 0.07 (*n* = 152) using HEPES.

Chromatography was performed using a Prominence Modular LC-20 Series (Shimadzu, Duisburg, Germany) equipped with a photodiode array detector (SPD M30, detection wavelength 240 nm) as previously described (15). The HPLC method was validated following the ICH guideline M10 on bioanalytical method validation and study sample analysis (41). Based on in-process QC samples (high/low), the CV of the intra-assay precision in serum regarding the determination of total vancomycin was 2.6/5.3%; the CV of the inter-assay precision was 3.5/5.0%; and the accuracy was 100.3/100.1%. The values in saline for the determination of vancomycin in ultrafiltrate were 1.3/2.8%, 2.4/5.3%, and 100.0/97.9%. To validate the total procedure of the determination of the unbound fraction (i.e., the ultrafiltration step plus the analytical step), a serum sample spiked with 25 mg/L vancomycin was processed with each run (38). The unbound fraction in this QC sample was 69.6 ± 1.7% (CV 2.4%) in phosphate-buffered serum and 66.7 ± 2.9% (CV 4.4%) in HEPES-buffered serum. Stability of total and free vancomycin was analyzed in pooled serum of two healthy volunteers spiked with 25 (high) or 5m/L (low) vancomycin. Analyzed conditions included one to three freeze-thaw cycles (with storage for 24 h at −20°C in between each freeze-thaw cycle) as well as storage for 6 days in the refrigerator (4–8°C) with one subsequent freeze-thaw cycle (*n* = 3 + 3 [high + low] for each condition). Total and free concentrations remained stable in all conditions with a mean (± SD) unbound fraction of 72.5 ± 1.8% (*n* = 30; see Table S3).

### Statistical analysis

Descriptive statistics and linear regression analyses were performed with GraphPad Prism 10 (GraphPad Software, La Jolla, CA, USA) as pooled analyses of all observations. Biochemical parameters were matched to the temporally closest vancomycin plasma concentration measurement, with a maximum allowable interval of 2 days between paired values. Continuous variables are reported as mean ± standard deviation (SD), unless otherwise specified. Comparisons between groups were made using the Mann-Whitney *U* test. A *P*-value < 0.05 was considered statistically significant.

A random-intercept linear mixed-effects model for the unbound fraction of vancomycin was developed in R project 4.5.1 (R Foundation for Statistical Computing, Vienna, Austria). Inter-individual variability was modeled as a random effect (subject ID) to account for correlation among longitudinal measures within the same subject. Inter-occasion variability was modeled as random effect nested within subject ID, where distinct occasions were defined by time gaps between consecutive longitudinal measurements. Multiple gap cut-offs (1–20 days) were evaluated. A 3-day cut-off was selected for the final model, as it yielded the highest proportion of variance explained by the cluster random effect. Fixed effects included age, sex, ward specialty, plasma concentrations of creatinine, total bilirubin, albumin, total protein, C-reactive protein, leukocytes, hemoglobin, and total vancomycin as complete case analysis. In addition, the type of buffer solution (HEPES or phosphate)—a methodological parameter of the analytical assay that was changed during the study period (cf. Limitations)—was included as another fixed effect. Non-significant fixed effects were iteratively removed to derive the final model. For categorical predictors, overall group effects were tested by F-tests (Satterthwaite’s method), and, when significant, pairwise comparisons of estimated marginal means were conducted with correction for multiple testing (Tukey-adjusted).

## Data Availability

Vancomycin plasma concentrations as measured by HPLC (total and free concentrations) and immunoassay (total concentrations), respectively. This dataset was used in the following research project: https://github.com/adejacoukr/VAN-PPB.

## AFTER EPUB

*[This article was published on 26 January 2026 with an error in Fig. 2 and its caption. Figure 2 and its caption were corrected in the current version, posted on 2 February 2026.]*

## SUPPLEMENTAL MATERIAL

Supplemental material aac.01593-25-s0001.docx Fig. S1 to S6; Tables S1 to S3.

ASM does not own the copyrights to Supplemental Material that may be linked to, or accessed through, an article. The authors have granted ASM a non-exclusive, world-wide license to publish the Supplemental Material files. Please contact the corresponding author directly for reuse.
