# Supplementary material for: Protein binding of vancomycin in a large mixed patient population at a university hospital
Source: Antimicrob Agents Chemother. 2026 Jan 26;70(3):e01593-25. doi: 10.1128/aac.01593-25 (PMC12959147; doi:10.1128/aac.01593-25)
Supplement: Supplemental material — Fig. S1 to S6; Tables S1 to S3. [file aac.01593-25-s0001.docx]

**Protein binding of vancomycin in a large mixed patient population at a university hospital**

Alexander DEJACO^1^#, Constantin LIER^2^, Sabrina KRAUTBAUER^3^, Frieder KEES^4^, Christoph DORN^2^, Martin G. KEES^1^

^1)^ Department of Anaesthesiology, University Hospital Regensburg, Regensburg, Germany

^2)^ Department of Pharmaceutical and Medicinal Chemistry I, University of Regensburg, Regensburg, Germany

^3)^ Institute of Clinical Chemistry and Laboratory Medicine, University Hospital Regensburg, Regensburg, Germany

^4)^ Department of Pharmacology, University of Regensburg, Regensburg, Germany

Running head: Vancomycin protein binding in a large cohort

#Address correspondence to Alexander Dejaco, alexander.dejaco@ukr.de

**APPENDIX**

| **Supplementary Figure S1:** Sorted heatmap showing available covariate data (cyan) and missing values (red). One row per observation (n=706). |
| --- |
| 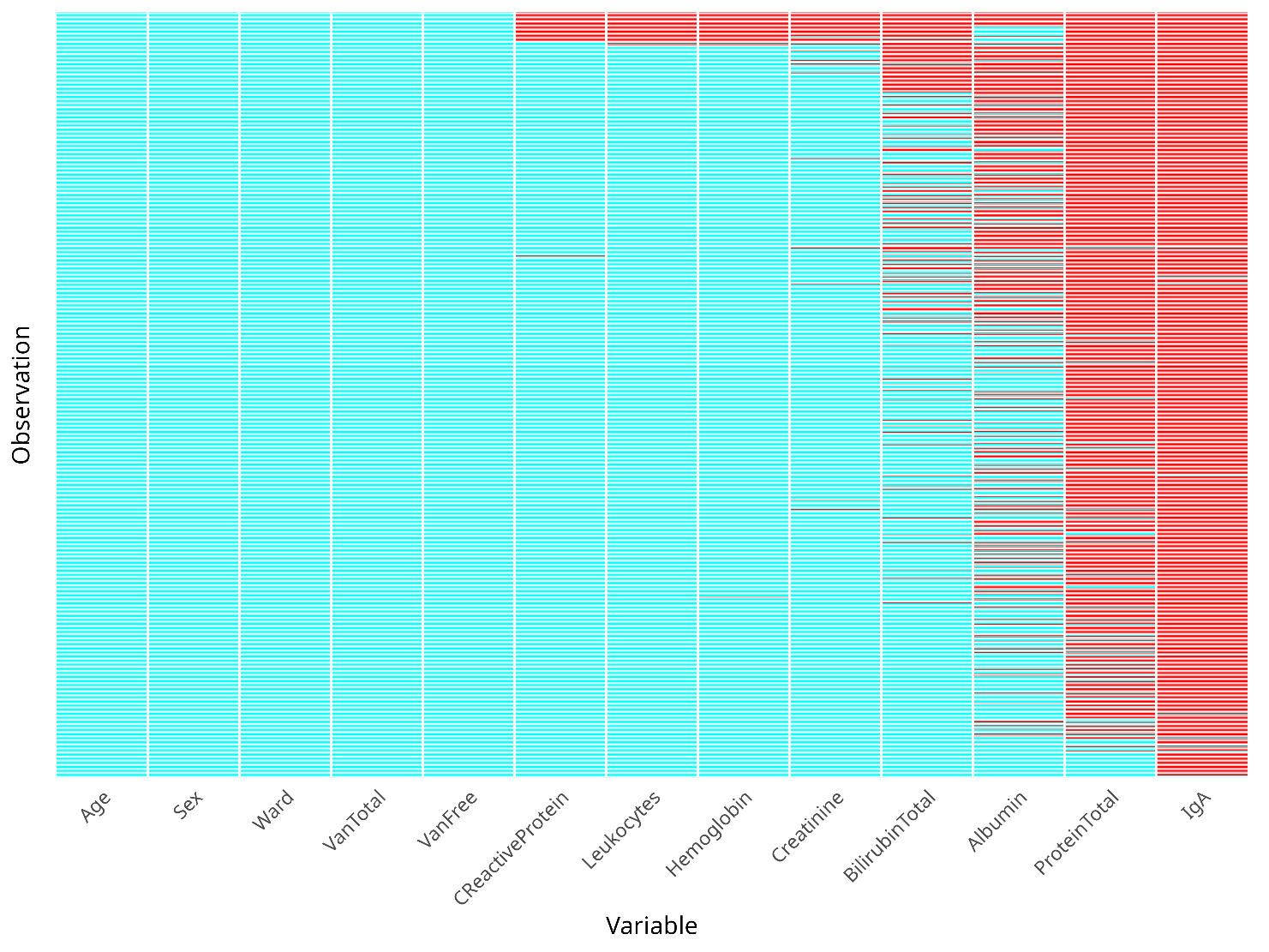 |

| **Supplementary Figure S2:** Variation of unbound vancomycin fraction (*f*_u_). Individual data and mean from patients where 4 or more samples were available over a sampling period of 22 to 68 days. |
| --- |
| 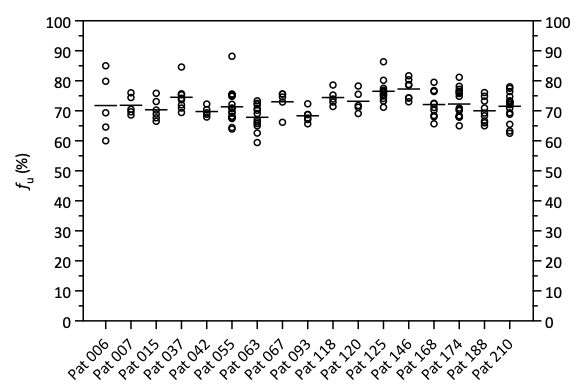 |

| **Supplementary Figure S3:** Variation of unbound vancomycin fraction (*f*_u_) over time in patients where 4 or more samples were available over a sampling period of 22 to 68 days. | |
| --- | --- |
| 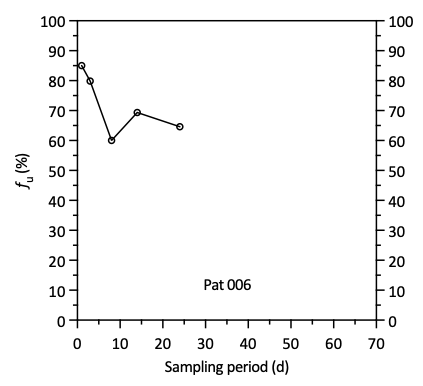 | 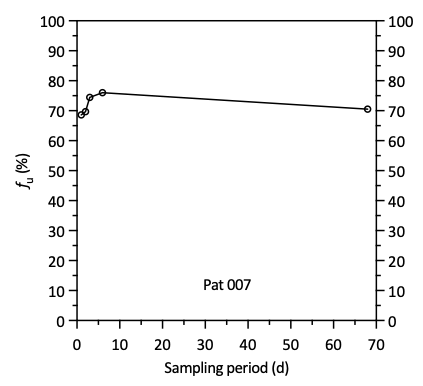 |
| 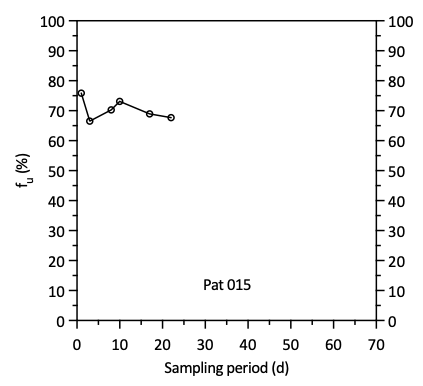 | 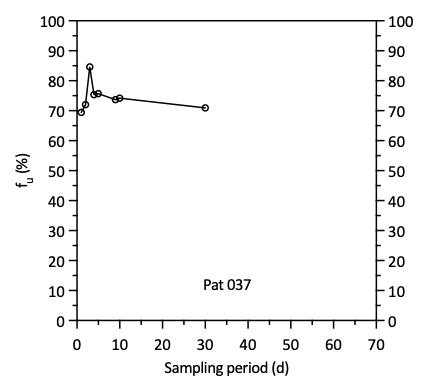 |
| 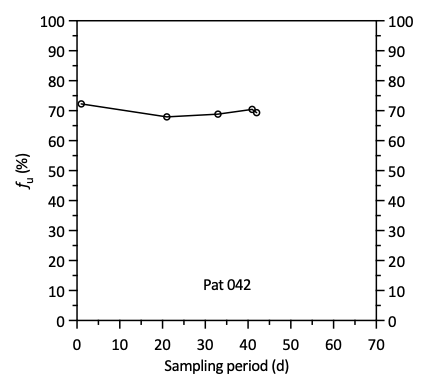 | 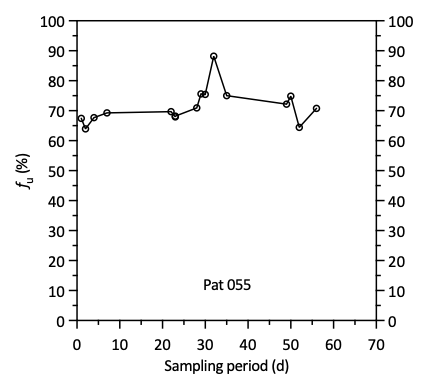 |
| 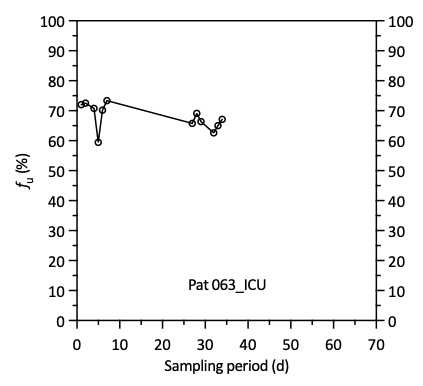 | 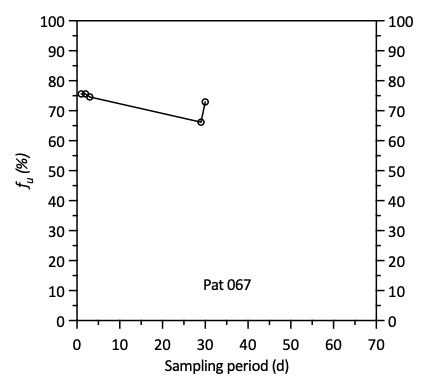 |
| 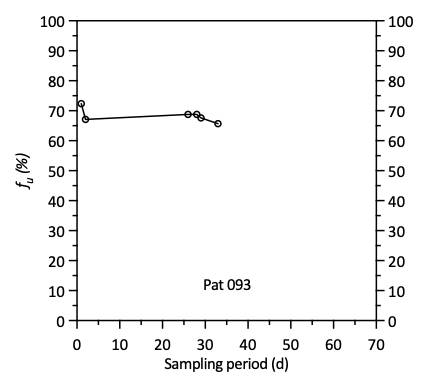 | 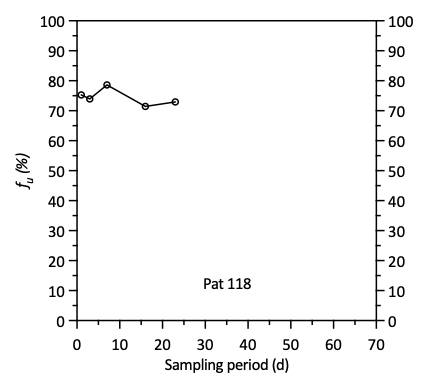 |
| 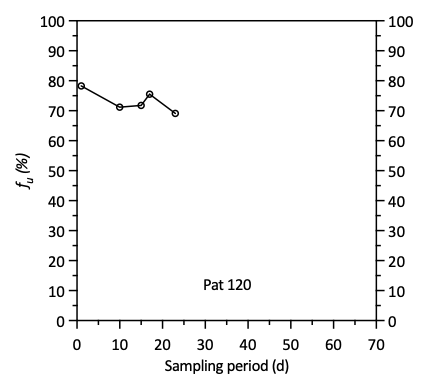 | 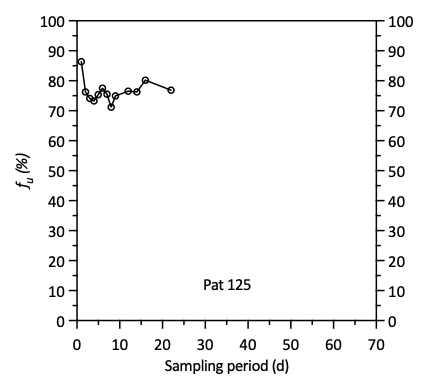 |
| 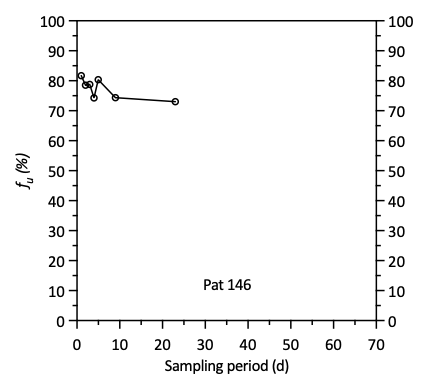 | 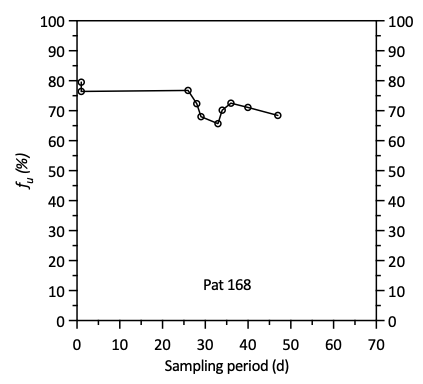 |
| 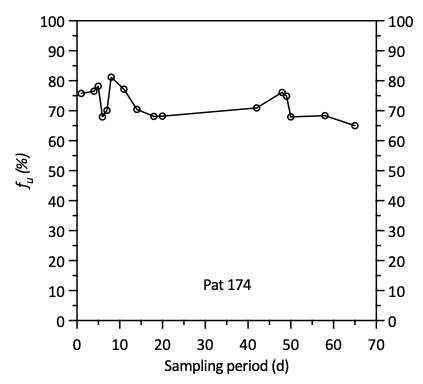 | 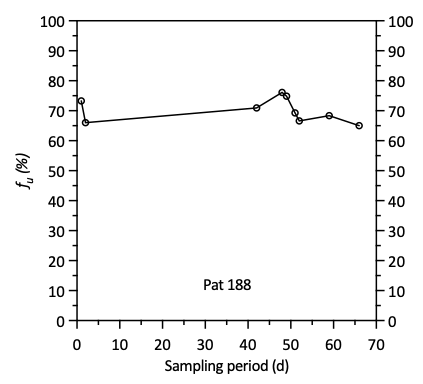 |
| 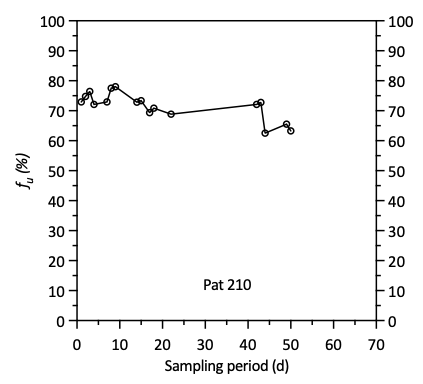 |  |

| **Supplementary Figure S4:** Boxplots of observed unbound vancomycin fractions (*f*_u_) across primary ward specialty (706 observations from 228 individuals). |
| --- |
| 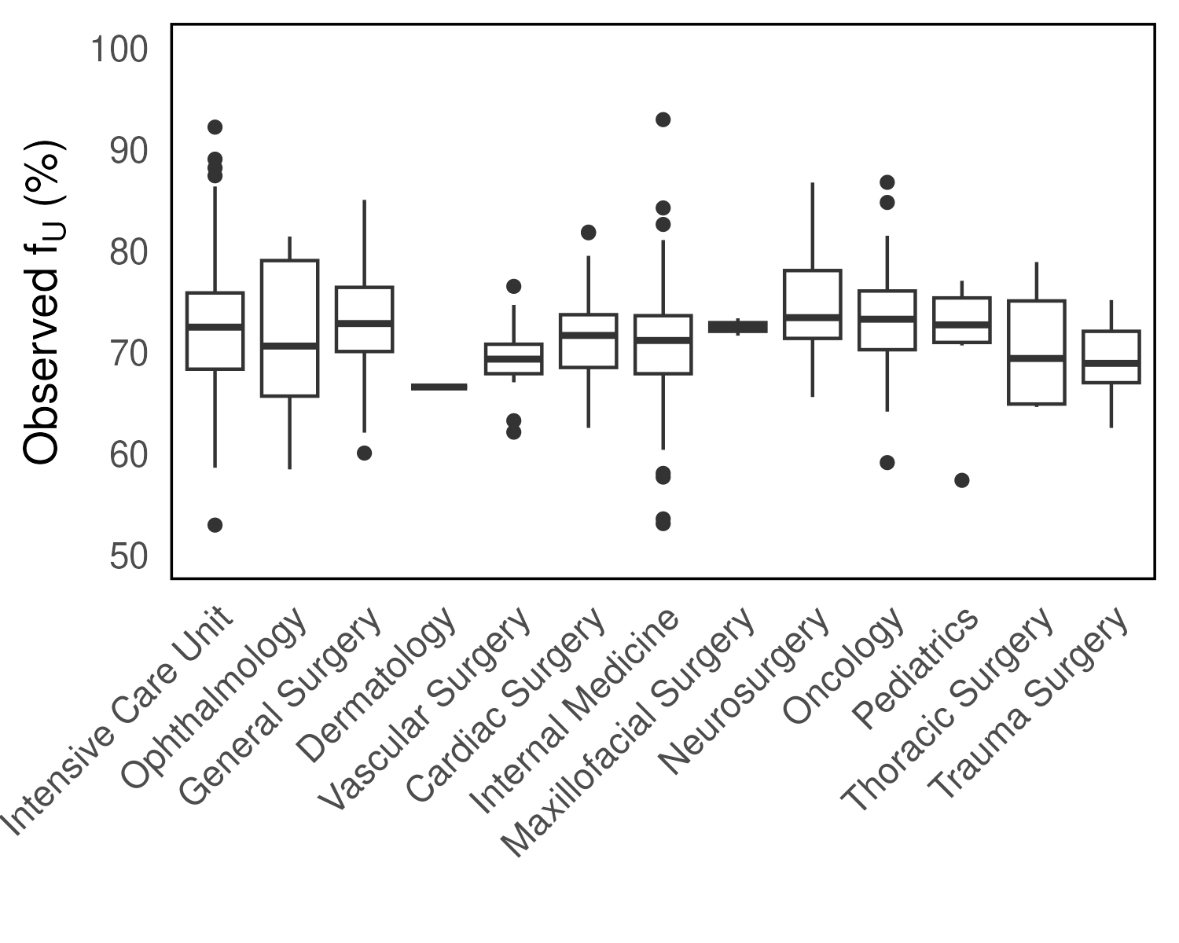 |

| **Supplementary Figure S5:** Model-predicted unbound vancomycin fractions (mean *f*_u_ ± 95% confidence interval) across primary ward specialty, as estimated by the linear mixed-effects model. |
| --- |
| 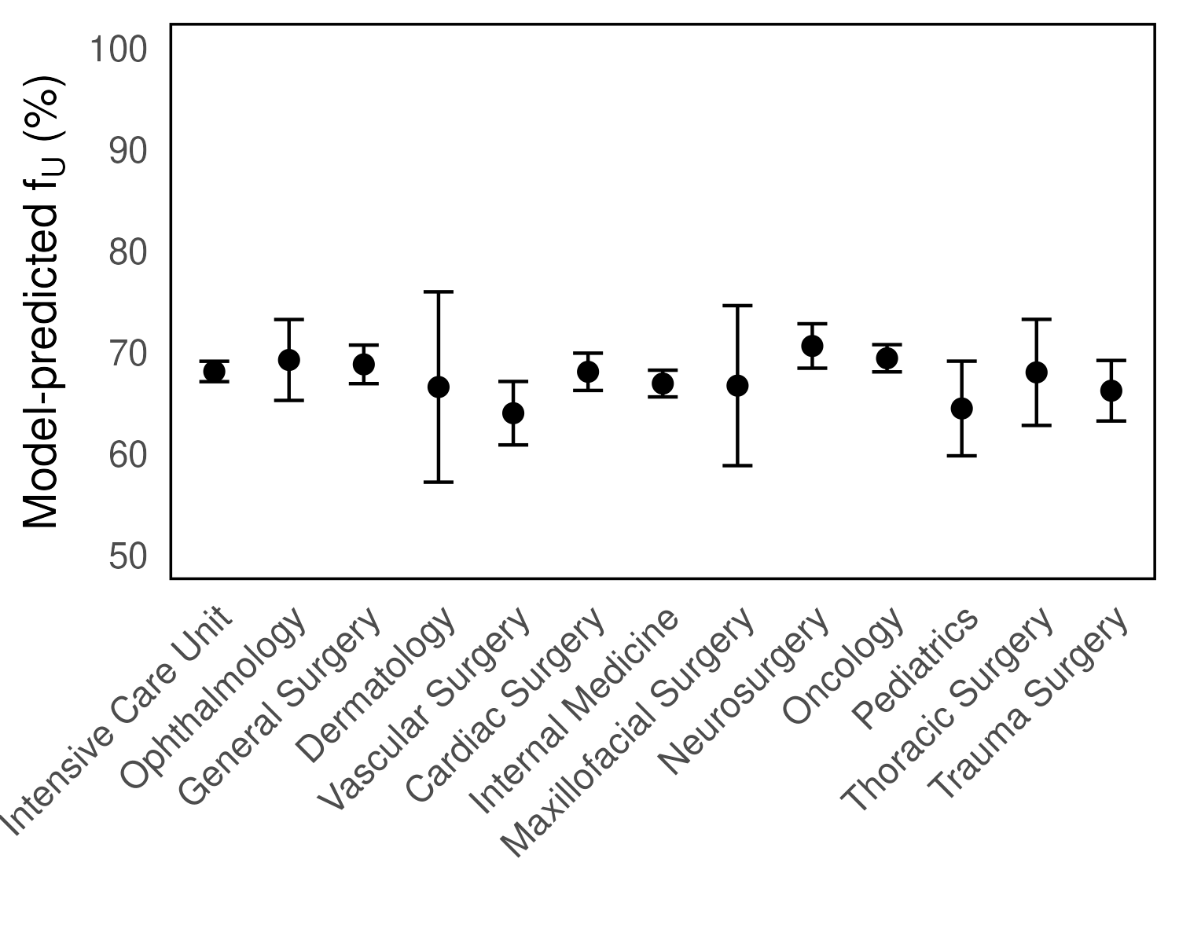 |

| **Supplementary Figure S6:** Correlation between the total vancomycin plasma concentration as determined by high-performance liquid chromatography (HPLC) and by the routine clinical laboratory assay (VANC3 immunoassay based on the kinetic interaction of microparticles in a solution [KIMS]). |
| --- |
|  |

**Supplementary Table S1: Clinical specialties in the dataset with counts (measurements)**

- Intensive Care Medicine (ICU): 330
- Internal Medicine (MED): 108
- Oncology (ONKO): 99
- Cardiothoracic Surgery (HTC): 56
- General Surgery (CHR): 34
- Neurosurgery (NCH): 31
- Trauma Surgery (UCH): 14
- Vascular Surgery (GCH): 14
- Ophthalmology (AUG): 7
- Pediatrics (PED): 6
- Thoracic Surgery (TCH): 4
- Oral & Maxillofacial Surgery (MKG): 2
- Dermatology (DERMA): 1

**Supplementary Table S2: Model specifications and full model results**

**Model 1 – full linear mixed effects model**

**Formula:** FU ~ (1 | PID/CLUSTER) + BILIG + LEUCO + BUFFER + ALB + AGE +

SEX + VANTOT + STATION + CRP + HB + CREA

**Random effects:**

Groups Name Variance Std.Dev.

CLUSTER:PID (Intercept) 4.729 2.175

PID (Intercept) 4.387 2.095

Residual 13.707 3.702

Number of obs: 284, groups: CLUSTER:PID, 189; PID, 138

**Fixed effects:**

Estimate Std. Error df t value Pr(>|t|)

(Intercept) 68.339986 4.400918 213.977603 15.529 <2e-16

BILIG -0.014531 0.075694 249.661409 -0.192 0.8479

LEUCO 0.009816 0.039790 218.286535 0.247 0.8054

BUFFERP 6.115586 0.667838 255.267662 9.157 <2e-16

ALB 0.041259 0.064339 183.495310 0.641 0.5221

AGE -0.011817 0.024898 97.875697 -0.475 0.6361

SEX1 -0.459526 0.782497 90.349959 -0.587 0.5585

VANTOT 0.019846 0.038199 244.818994 0.520 0.6038

STATIONGCH -11.088774 4.544387 223.549779 -2.440 0.0155

STATIONHTC -0.867278 3.189175 185.207733 -0.272 0.7860

STATIONICU -1.399900 2.892377 219.677335 -0.484 0.6289

STATIONMED -2.555276 2.993294 217.831364 -0.854 0.3942

STATIONMKG -2.772571 5.698986 221.642292 -0.487 0.6271

STATIONNCH -0.121345 4.266922 196.754758 -0.028 0.9773

STATIONOnko -0.589753 2.954578 214.976372 -0.200 0.8420

STATIONPED -8.246043 4.224928 157.325011 -1.952 0.0527

STATIONUCH -2.729084 5.644041 221.692709 -0.484 0.6292

CRP 0.004062 0.003509 223.797767 1.158 0.2482

HB 0.008802 0.186474 248.771484 0.047 0.9624

CREA -0.068316 0.248732 223.243637 -0.275 0.7838

**Model 2 – simpler linear mixed effects model and analysis of variance**

**Formula:** FU ~ (1 | PID/CLUSTER) + BUFFER + STATION

**Random effects:**

Groups Name Variance Std.Dev.

CLUSTER:PID (Intercept) 6.753 2.599

PID (Intercept) 2.301 1.517

Residual 13.829 3.719

Number of obs: 706, groups: CLUSTER:PID, 330; PID, 228

**Fixed effects:**

Estimate Std. Error df t value Pr(>|t|)

(Intercept) 68.09420 0.51322 289.97569 132.681 <2e-16

BUFFERP 5.78971 0.45235 578.05795 12.799 <2e-16

STATIONAUG 1.13308 2.04430 408.80002 0.554 0.5797

STATIONCHR 0.69832 0.99209 179.55889 0.704 0.4824

STATIONDERMA -1.53583 4.81099 529.34385 -0.319 0.7497

STATIONGCH -4.11993 1.58586 149.58392 -2.598 0.0103

STATIONHTC -0.03541 0.98143 90.08830 -0.036 0.9713

STATIONMED -1.19435 0.70164 190.37442 -1.702 0.0903

STATIONMKG -1.39991 4.01576 308.38748 -0.349 0.7276

STATIONNCH 2.51375 1.14157 207.58871 2.202 0.0288

STATIONOnko 1.30060 0.72355 156.02554 1.798 0.0742

STATIONPED -3.65476 2.36907 155.55770 -1.543 0.1249

STATIONTCH -0.09179 2.68223 272.63658 -0.034 0.9727

STATIONUCH -1.90263 1.55234 294.09543 -1.226 0.2213

**Post-hoc pair-wise analysis**

STATION emmean SE df lower.CL upper.CL

ICU 71.0 0.402 179.0 70.2 71.8

AUG 72.1 2.010 392.2 68.2 76.1

CHR 71.7 0.925 200.4 69.9 73.5

DERMA 69.5 4.790 513.9 60.0 78.9

GCH 66.9 1.560 160.2 63.8 69.9

HTC 71.0 0.912 86.2 69.1 72.8

MED 69.8 0.599 210.2 68.6 71.0

MKG 69.6 4.000 283.1 61.7 77.5

NCH 73.5 1.080 213.5 71.4 75.6

Onko 72.3 0.621 159.4 71.1 73.5

PED 67.3 2.350 166.8 62.7 72.0

TCH 70.9 2.660 277.6 65.7 76.1

UCH 69.1 1.500 286.5 66.1 72.0

$contrasts

contrast estimate SE df t.ratio p.value

ICU - AUG -1.1331 2.050 384.0 -0.554 1.0000

ICU - CHR -0.6983 0.998 190.7 -0.700 1.0000

ICU - DERMA 1.5358 4.810 511.4 0.319 1.0000

ICU - GCH 4.1199 1.600 157.7 2.583 0.3297

ICU - HTC 0.0354 0.992 98.2 0.036 1.0000

ICU - MED 1.1944 0.705 190.6 1.694 0.8947

ICU - MKG 1.3999 4.020 281.6 0.349 1.0000

ICU - NCH -2.5137 1.150 205.5 -2.194 0.5966

ICU - Onko -1.3006 0.727 161.0 -1.788 0.8529

ICU - PED 3.6548 2.370 165.3 1.541 0.9444

ICU - TCH 0.0918 2.690 274.2 0.034 1.0000

ICU - UCH 1.9026 1.550 278.4 1.224 0.9914

AUG - CHR 0.4348 2.210 371.0 0.197 1.0000

AUG - DERMA 2.6689 5.200 494.3 0.513 1.0000

AUG - GCH 5.2530 2.540 299.0 2.070 0.6847

AUG - HTC 1.1685 2.210 325.1 0.529 1.0000

AUG - MED 2.3274 2.090 378.7 1.111 0.9965

AUG - MKG 2.5330 4.470 302.3 0.566 1.0000

AUG - NCH -1.3807 2.280 350.0 -0.605 1.0000

AUG - Onko -0.1675 2.100 373.4 -0.080 1.0000

AUG - PED 4.7878 3.090 253.4 1.552 0.9424

AUG - TCH 1.2249 3.330 324.9 0.368 1.0000

AUG - UCH 3.0357 2.510 352.5 1.209 0.9924

CHR - DERMA 2.2342 4.880 505.0 0.458 1.0000

CHR - GCH 4.8183 1.800 166.1 2.674 0.2765

CHR - HTC 0.7337 1.300 129.1 0.566 1.0000

CHR - MED 1.8927 1.090 200.8 1.731 0.8796

CHR - MKG 2.0982 4.100 284.2 0.511 1.0000

CHR - NCH -1.8154 1.420 209.6 -1.280 0.9872

CHR - Onko -0.6023 1.110 184.0 -0.544 1.0000

CHR - PED 4.3531 2.520 168.8 1.730 0.8794

CHR - TCH 0.7901 2.810 268.6 0.281 1.0000

CHR - UCH 2.6010 1.760 272.2 1.474 0.9606

DERMA - GCH 2.5841 5.050 475.1 0.512 1.0000

DERMA - HTC -1.5004 4.880 493.1 -0.308 1.0000

DERMA - MED -0.3415 4.830 508.6 -0.071 1.0000

DERMA - MKG -0.1359 6.250 406.2 -0.022 1.0000

DERMA - NCH -4.0496 4.910 496.8 -0.824 0.9998

DERMA - Onko -2.8364 4.830 507.5 -0.587 1.0000

DERMA - PED 2.1189 5.340 435.4 0.397 1.0000

DERMA - TCH -1.4440 5.480 456.9 -0.264 1.0000

DERMA - UCH 0.3668 5.020 490.4 0.073 1.0000

GCH - HTC -4.0845 1.800 136.8 -2.267 0.5448

GCH - MED -2.9256 1.660 163.6 -1.765 0.8639

GCH - MKG -2.7200 4.280 275.0 -0.635 1.0000

GCH - NCH -6.6337 1.890 175.4 -3.512 0.0314

GCH - Onko -5.4205 1.670 158.7 -3.249 0.0696

GCH - PED -0.4652 2.800 162.4 -0.166 1.0000

GCH - TCH -4.0281 3.080 240.1 -1.309 0.9847

GCH - UCH -2.2173 2.160 219.5 -1.025 0.9983

HTC - MED 1.1589 1.090 112.9 1.065 0.9974

HTC - MKG 1.3645 4.100 276.6 0.333 1.0000

HTC - NCH -2.5492 1.410 147.2 -1.805 0.8443

HTC - Onko -1.3360 1.100 104.8 -1.213 0.9914

HTC - PED 3.6193 2.520 152.8 1.438 0.9665

HTC - TCH 0.0564 2.810 243.6 0.020 1.0000

HTC - UCH 1.8672 1.760 215.2 1.062 0.9976

MED - MKG 0.2056 4.040 282.0 0.051 1.0000

MED - NCH -3.7081 1.230 210.7 -3.015 0.1262

MED - Onko -2.4949 0.854 180.5 -2.922 0.1601

MED - PED 2.4604 2.410 168.1 1.019 0.9984

MED - TCH -1.1026 2.720 273.4 -0.405 1.0000

MED - UCH 0.7083 1.620 276.4 0.438 1.0000

MKG - NCH -3.9137 4.140 280.2 -0.945 0.9993

MKG - Onko -2.7005 4.050 281.4 -0.667 1.0000

MKG - PED 2.2549 4.630 268.1 0.487 1.0000

MKG - TCH -1.3081 4.800 293.2 -0.272 1.0000

MKG - UCH 0.5027 4.270 284.4 0.118 1.0000

NCH - Onko 1.2132 1.240 198.5 0.977 0.9989

NCH - PED 6.1685 2.580 174.2 2.392 0.4546

NCH - TCH 2.6055 2.870 267.0 0.909 0.9995

NCH - UCH 4.4164 1.850 261.1 2.386 0.4572

Onko - PED 4.9554 2.420 165.6 2.046 0.7010

Onko - TCH 1.3924 2.730 269.3 0.511 1.0000

Onko - UCH 3.2032 1.630 268.1 1.970 0.7513

PED - TCH -3.5630 3.540 221.4 -1.005 0.9986

PED - UCH -1.7521 2.790 201.0 -0.629 1.0000

TCH - UCH 1.8108 3.050 282.9 0.593 1.0000

**Supplementary Table S3:** Stability (relative recovery compared to fresh samples, %) of total and free concentrations of vancomycin in pooled serum from two healthy volunteers (vancomycin concentrations: high 25 mg/L, low 5 mg/L).

|  | Fresh samples | 24 h at -20 °C 1× freeze/thaw | 2× 24 h at -20 °C 2× freeze/thaw | 3× 24 h at -20 °C 3× freeze/thaw | 6 days at 4-8 °C 1x freeze/thaw |
| --- | --- | --- | --- | --- | --- |
| Stability (%) of total vancomycin concentrations | | | | | |
| high | 99,7 | 99,0 | 97,7 | 99,1 | 100,6 |
|  | 100,3 | 99,5 | 99,5 | 99,6 | 100,6 |
|  | 100,0 | 101,8 | 99,2 | 101,5 | 99,9 |
| low | 100,5 | 101,0 | 101,6 | 99,6 | 100,0 |
|  | 97,9 | 92,1 | 99,3 | 99,2 | 101,6 |
|  | 101,6 | 99,4 | 103,7 | 98,8 | 102,6 |
| mean | **100,0** | **98,8** | **100,2** | **99,6** | **100,9** |
| SD | 1,2 | 3,4 | 2,1 | 1,0 | 1,0 |
| Stability (%) of free vancomycin concentrations | | | | | |
| high | 99,9 | 99,5 | 100,0 | 98,9 | 98,6 |
|  | 100,3 | 99,4 | 99,9 | 98,9 | 98,9 |
|  | 99,9 | 99,0 | 99,1 | 99,6 | 97,9 |
| low | 100,2 | 99,7 | 97,2 | 96,5 | 95,6 |
|  | 100,7 | 101,3 | 98,4 | 96,5 | 98,9 |
|  | 99,2 | 98,8 | 98,6 | 100,5 | 94,4 |
| mean | **100,0** | **99,6** | **98,9** | **98,5** | **97,4** |
| SD | 0,5 | 0,9 | 1,1 | 1,6 | 1,9 |
| Unbound fraction (%) | | | | | |
| mean | **73,4** | **73,1** | **72,5** | **72,3** | **71,4** |
| SD | 1,921 | 2,2 | 1,3 | 1,7 | 1,5 |

Abbr.: SD standard deviation
